# Seven-Year Analysis of Microbial Keratitis Tendency at an Ophthalmology Department in Poland: A Single-Center Study

**DOI:** 10.1155/2020/8851570

**Published:** 2020-10-28

**Authors:** Klaudia Ulfik, Sławomir Teper, Michał Dembski, Anna Nowińska, Ewa Wróblewska-Czajka, Edward Wylęgała

**Affiliations:** ^1^Faculty of Medical Sciences in Zabrze, Medical University of Silesia, Chair and Department of Ophthalmology, Panewnicka 65, 40-760 Katowice, Poland; ^2^District Railway Hospital, Panewnicka 65, 40-760 Katowice, Poland

## Abstract

This study aimed to analyze the frequency, drug susceptibility, and drug resistance of pathogens causing microbial keratitis (a corneal inflammation) in the Clinical Department of Ophthalmology, Medical University of Silesia, Katowice. Despite intensive treatment, severe inflammation causes irreversible blindness in ∼7% of cases and eye loss (evisceration or enucleation of the eyeball) in ∼1% of cases at our hospital. The choice of a targeted drug depends on the culture result and drug resistance of the microorganism. This was a retrospective observation study. Conjunctival swabs and corneal scrapes were collected between January 1, 2013, and December 31, 2019, in the tertiary reference center for keratitis. The collected data included the type of material received, culture result, and antimicrobial susceptibilities. Of the 2482 samples analyzed, 679 were positive and 1803 were negative. Of the total pathogens isolated, 69.9% were Gram-positive bacteria, 20.8% were Gram-negative bacteria, and 7.1% were fungi. A significant increase in the number of Gram-positive methicillin-resistant *Staphylococcus aureus* and a partial increase in the number of Gram-negative beta-lactams-resistant bacteria were observed. All fungal species were sensitive to amphotericin B, 82.81% were sensitive to voriconazole, and 56.25% were sensitive to fluconazole. Dual drug therapy (levofloxacin and tobramycin) was the first-line treatment. Drug susceptibility testing of the cultured microorganisms is necessary to initiate targeted treatment. Increased drug resistance was observed in this study. In the present study, most bacteria were sensitive to fluoroquinolones. *Ciprofloxacin* therapy remains the recommended empirical treatment in microbial keratitis. According to our study, voriconazole remains a first-line antifungal drug, when a fungal infection is suspected.

## 1. Introduction

Keratitis is a corneal inflammation and has two main types: infectious and noninfectious. Infectious keratitis includes bacterial, viral, fungal, and protozoal inflammation, while noninfectious keratitis includes many diseases caused by an abnormal immunological reaction or disturbed physiological processes on the eye surface [1].

Inflammatory corneal diseases remain a major challenge in ophthalmology. Microbial keratitis remains a serious cause of corneal opacification and vision loss worldwide [2]. Infectious keratitis can lead to vision loss. Bacterial keratitis is a potentially devastating ocular infection [3]. Risk factors for bacterial keratitis are wearing contact lens, ocular surface diseases, ocular trauma, reduced immunity, and prior ocular surgery. A significant number of patients with keratitis lose their eyesight, and evisceration or enucleation of the eyeball is necessary in cases with blind painful eyes or in cases in which adjacent tissues are endangered by progressive infection from a blind inflamed eye [2, 4].

The rapid initiation of empirical and then targeted treatment is crucial. The choice of a targeted drug depends on the culture result and drug resistance of the cultured microorganism. The increasing drug resistance of microorganisms is an important public health problem worldwide [2]. The highly frequent prescription of ophthalmic antibiotic drops, which are mainly broad-spectrum agents, is also responsible for this drug resistance.

It is vital to establish both global and local trends in eye infections in order to determine the most effective treatment, especially at the initial stage when the treatment is not based on culture results.

The present study aimed to analyze the frequency, drug susceptibility, and drug resistance of pathogens causing microbial keratitis over a 7-year period in the Clinical Department of Ophthalmology, Medical University of Silesia, Katowice. Corneal transplantation in our clinic is also performed in patients not responding to conservative treatment. More than 200 patients undergo corneal transplantation annually in the clinic. One-fourth of these transplants are performed urgently due to acute keratitis and its complications, e.g., corneal perforation. Urgent corneal transplantations are mainly required in cases with fungal keratitis. Despite intensive treatment, severe inflammation results in irreversible blindness in about 7% of cases in our hospital. Moreover, inflammation results in eye loss (evisceration or enucleation of the eyeball) in about 1% of cases.

## 2. Materials and Methods

Conjunctival swabs and corneal scrapes were collected by ophthalmologists between January 1, 2013, and December 31, 2019, in our center. The mean age of the patients was 58 years. Patients who presented with the corneal ulcer (epithelial defect and stromal inflammation) were included to the study. Exclusion criteria were pregnancy, age under 18, no consent to collect corneal scrapes, impending corneal perforation, or descemetocele. The results were obtained from the hospital database. The collected data included the type of material received, culture result, and antimicrobial susceptibilities.

Samples were collected from the conjunctival sac using swabs with 0.9% NaCl. Moreover, corneal scrapes were collected using sterile 23 *G* needles. The samples were kept in a microbiological transport kit at 37°C and transported to the microbiological laboratory. The samples were then cultured on two blood agar plates, one chocolate agar plate, and one Sabouraud agar plate (bioMerieux). The Sabouraud agar plate was used to detect fungal infections. The agar plates were incubated in aerobic and anaerobic conditions. The blood and chocolate agar plates were incubated for at least 5 days, while the Sabouraud agar plate was incubated for at least 7 days. All the agars were obtained from one company. In rare, clinically justified cases with a strong suspicion of *Acanthamoeba* infection, a nonnutrient agar seeded with *Escherichia coli* for *Acanthamoeba* isolation was used. Confocal microscopy has also been performed as a noninvasive useful method to diagnose *Acanthamoeba* keratitis and to obtain earlier diagnosis. The isolation technique remained consistent throughout the study period. *Candida* chromogenic agar (CHROMagar) was used to distinguish *Candida* species. The sensitivity of bacteria to antibiotics was determined by the disc-diffusion method EUCAST (European Committee on Antimicrobial Susceptibility Testing).

Significant isolates were tested against selected antibiotics in accordance with the local microbiological protocol. Every isolate was classified as antibiotic resistant or susceptible. *Staphylococcus aureus* isolates were tested in a standard manner to check for methicillin resistance. Cefoxitin-resistance isolates were tested for methicillin resistance. Enterobacteriaceae were classified as beta-lactams susceptible or resistant. All fungal isolates were tested for susceptibility to fluconazole, voriconazole, itraconazole, and amphotericin. The minimum inhibitory concentration was determined for about 15% of the antifungal agents.

**Figure 1:**
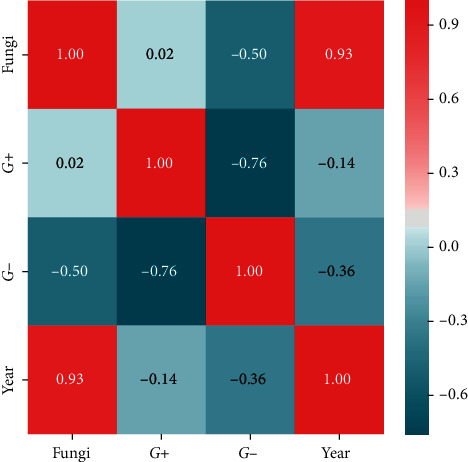
The matrix of correlations a.

**Figure 2:**
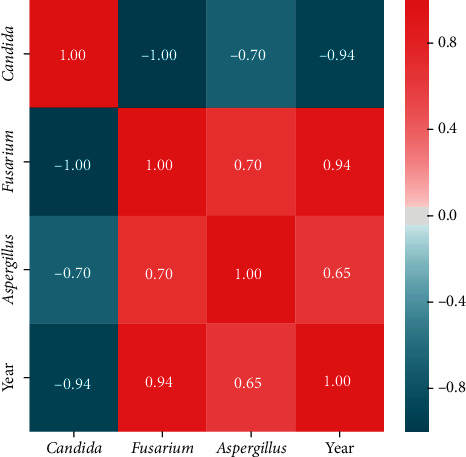
The matrix of correlations b.

**Figure 3:**
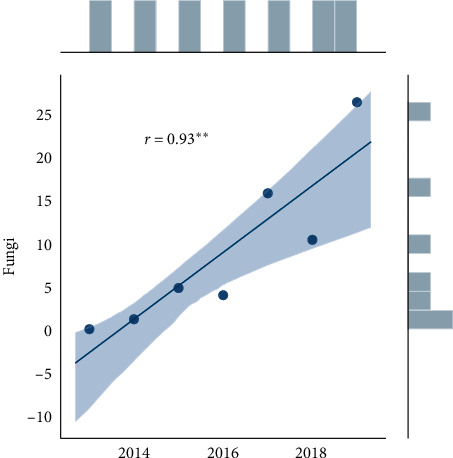
Spearman's correlation coefficients—Fungi.

**Figure 4:**
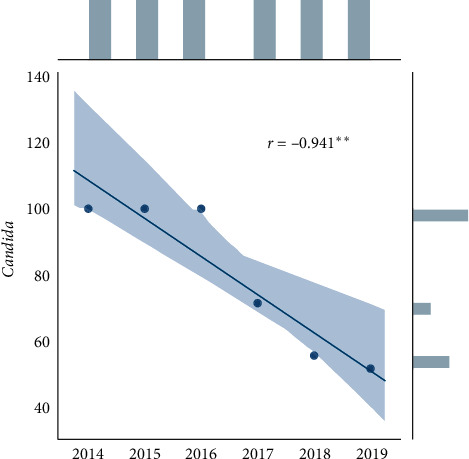
Spearman's correlation coefficients—*Candida*.

**Figure 5:**
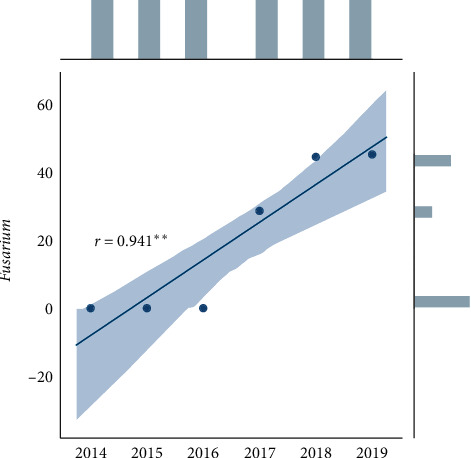
Spearman's correlation coefficients—*Fusarium*.

**Figure 6:**
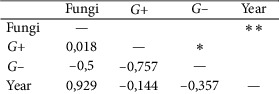
Statistical dependence between microorganism and year (*p* < 0.05 = single star, *p* < 0.01 = double star symbol, *p* < 0.001 = triple star).

**Figure 7:**
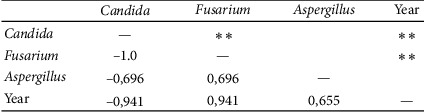
Statistical dependence between fungi and year.

**Figure 8:**
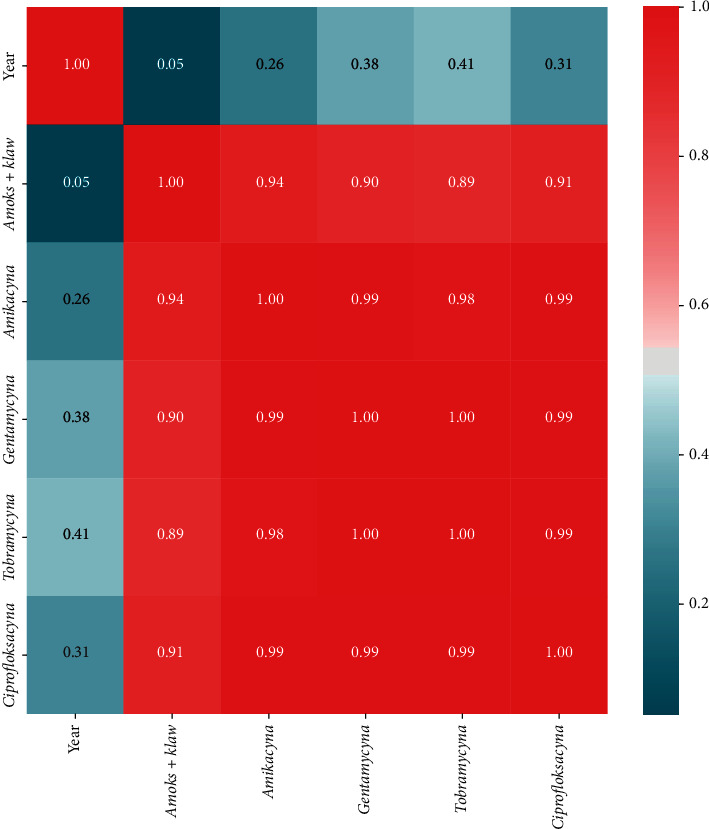
The matrix of Spearman's correlation coefficients between antibiotics.

Statistical analysis was performed using Microsoft Excel 2007 and Statistica (version 13.1. pl), Pandas, and Pingouin statistical packages dedicated for Python. A *p* value of <0.05 was considered statistically significant, with consideration made for multiple testing in the interpretation. The results of the normality test using the Shapiro–Wilk formula did not confirm the hypothesis regarding the normal distribution of most analyzed variables. In further analysis, nonparametric Spearman's correlation coefficients were used to study statistical dependence. This estimator is more resistant to outliers. To determine the strength of dependency between the variables, the matrix of correlations was analyzed (Figures 1 and 2). Graphs representing Spearman's correlation coefficients are provided in Figures 3–5. The relationship between the number of fungi and number of years was studied, and the number of fungi was found to significantly increase each year (*p* < 0.01, double star symbol). A strong negative correlation was found between the numbers of Gram-positive and Gram-negative bacteria (*p* < 0.05) (Figure 6). In the case of *Candida* and *Fusarium* spp., Spearman's correlation coefficients showed a completely opposite tendency (*p* < 0.001). A significant positive correlation was observed between *Fusarium* spp. and the number of years (*p* < 0.01), while a significant negative correlation was observed between *Candida* spp. and the number of years (*p* < 0.01) (Figure 7). With regard to the sensitivity of Gram-positive bacteria to antibiotics, the matrix of Spearman's correlation coefficients is shown in Figure 8.

## 3. Results

### 3.1. Trend Analysis

**Figure 9:**
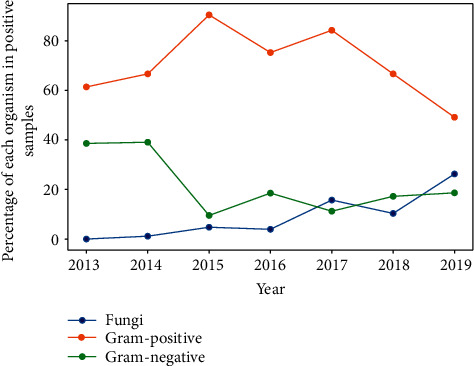
Percentage of each organism in positive samples.

In total, 2482 samples were analyzed during the study period. The mean age of the patients in the study was 58 ± 21 (SD). 1429 (57.57%) of the cases were men and 1053 (42.42%) were women. 20% of patients wear contact lenses. 236 (9.5%) of patients had in the past corneal graft. The number of tests performed regularly increased during this period. Of the samples analyzed, 679 (27.36%) were positive and 1803 (72.64%) were negative. The highest percentage of positive cultures (37.7%) was obtained in 2015. The number of positive cultures showed an upward trend. Of the total pathogens isolated, 69.9% were Gram-positive bacteria, 20.8% were Gram-negative bacteria, and 7.1% were fungi.  Figure 9 shows the trends of individual microorganisms.

In cases with *Acanthamoeba* keratitis, the clinical diagnosis was established on the basis of in vivo confocal microscopy and interviews; agar plates were rarely used.

**Figure 10:**
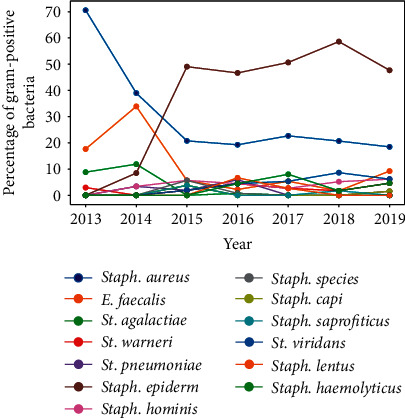
Percentage of gram positive bacteria.

**Figure 11:**
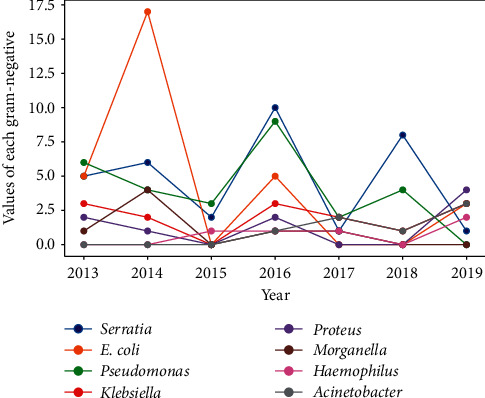
Percentage of gram negative bacteria.

A slight decreasing trend in the percentage of Gram-positive bacteria and a decreasing trend in the percentage of Gram-negative bacteria were noted. In the case of fungi, an increasing trend was noted (Figure 9). The most common Gram-positive bacteria were *S*. *epidermidis* (41.04%), *S*. *aureus* (26.04%), *S*. *hominis*, and *S*. *viridans* (4.16%) (Figure 10). The most common Gram-negative bacteria were *Serratia* spp. (24.81%), *E*. *coli* (22.55%), and *Pseudomonas aeruginosa* (21.05%) (Figure 11).

**Figure 12:**
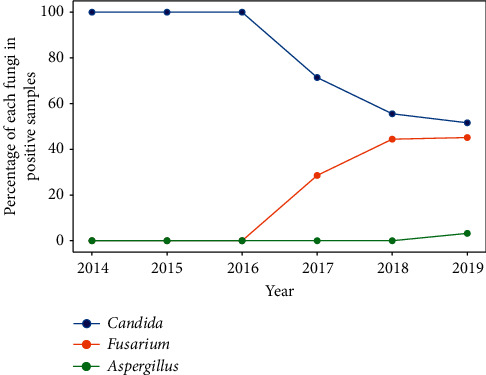
Percentage of each fungi in positive samples.

**Figure 13:**
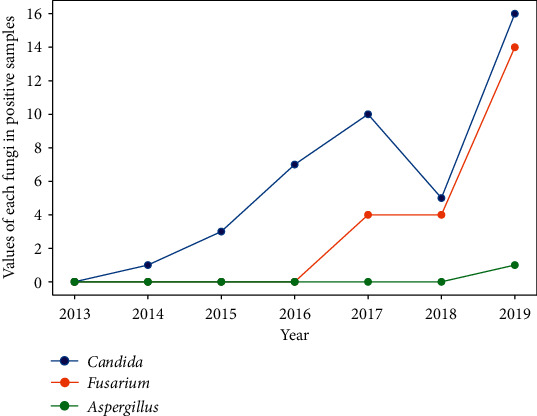
Values of each fungi in positive samples.

The most common fungal isolates belonged to *Candida* spp. (64.41%) and *Fusarium* spp. (33.84%); they accounted for 98.25% of the total fungal isolates. In 2019, a single species of *Aspergillus* was detected. In the case of fungi, an increasing trend and an increase in the variety of cultured fungi were observed (Figures 12 and 13).

### 3.2. Antimicrobial Susceptibilities

#### 3.2.1. Gram-Positive Bacteria

A significant increase in the number of Gram-positive methicillin-resistant *S*. *aureus* (MRSA) (from 4.34% to 57.40%) was observed, while vancomycin-resistant *S*. *aureus* (VRSA) was not detected. All MRSA (100%) isolates were susceptible to vancomycin. Individual cases of high-level aminoglycoside-resistant (HLAR+) *Enterococcus faecalis* were detected. 81.5% bacteria were susceptible to ofloxacin, 100% to vancomycin, and 90.1% to gentamicin. The sensitivity of Gram-positive bacteria to chloramphenicol and clindamycin is not reported, as not all bacteria were tested for susceptibility to these antibiotics. Gentamicin, vancomycin, and ofloxacin were found to be the most effective antibiotics for Gram-positive bacterial keratitis.

#### 3.2.2. Gram-Negative Bacteria

**Figure 14:**
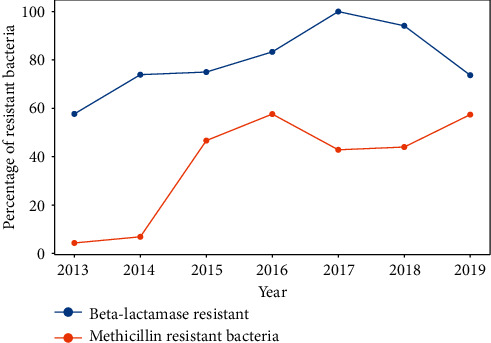
Percentage of resistant bacteria.

A slight increase in the number of Gram-negative (Enterobacteriaceae) beta-lactams-resistant bacteria was observed. Beta-lactams-resistant bacteria were more frequently detected than beta-lactams-sensitive bacteria throughout the study period (Figure 14). 94.8% bacteria were susceptible to gentamicin and 97.2% to ciprofloxacin.

#### 3.2.3. Fungi

**Figure 15:**
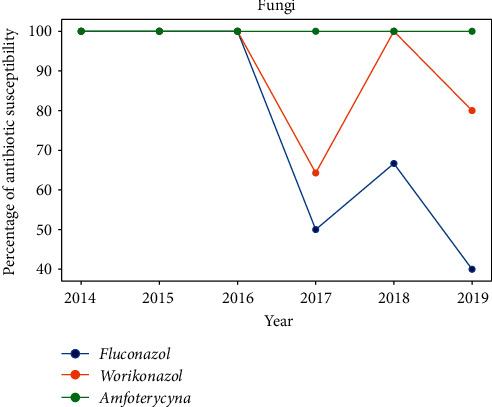
Percentage of antibiotic susceptibility.

All the fungal species were sensitive to amphotericin, 82.81% were sensitive to voriconazole, and 56.25% were sensitive to fluconazole. *A*. *fumigates* was only sensitive to amphotericin. All isolates of *C*. *glabrata* and *C*. *krusei* were naturally resistant to fluconazole. These fungi were isolated from the samples obtained in 2017, 2018, and 2019. All *Candida* species were sensitive to amphotericin B. Of the 14 *Fusarium* species tested for voriconazole susceptibility, four were found to be resistant. In the following years during the study period, the variety of cultivated fungi, their quantity, and their antifungal drug resistance increased (Figure 15).

## 4. Discussion

Gram-positive bacteria were the most commonly detected isolates in the present study (69.6%). The number of Gram-positive bacteria detected increased initially, followed by a plateau period and a subsequent decrease. The most commonly detected Gram-positive bacterium was *S*. *epidermidis* (41.04%). It is also the most frequently cultured pathogen in other ophthalmic centers [5]. Gram-positive cocci have also been found to be the most common causative agents of keratitis in a hospital in Japan [6]. Eye infection may also involve contamination of the physiological flora from the eyelid skin and eyelid margin, which does not cause keratitis. *S*. *epidermidis* was the most common isolate in the present study; however, we observed a reducing trend in its occurrence, with a shift toward an increasing trend in the occurrence of *S*. *aureus*. In Belgium, *Pseudomonas* was found to be the main causative agent of keratitis in a previous study; however, it is important to note that mainly contact lens wearers were examined in that study [7]. Few studies have reported the epidemiology of keratitis in Europe. Whether the study group wore contact lenses and what was the age of the study group are key points that should be considered.

Dual drug therapy involving levofloxacin (the third-generation fluoroquinolone) and tobramycin is the first-line treatment in our center. In the case of a high clinical suspicion of a fungal infection, voriconazole is empirically administered. The antibiotic therapy is immediately modified after receiving the results of microbiological tests. In patients with a strong suspicion of *Acanthamoeba* infection (e.g., those wearing contact lenses or swimming in water while wearing lenses), therapy with 0.1% propamidine isethionate (Brolene) and 0.02% chlorhexidine is initiated. Drug resistance results may vary in vitro and in vivo. In the present study, most bacteria were sensitive to fluoroquinolones. If the pathological factor was not detected and resistance to first-line treatment was known, gentamicin, moxifloxacin, and vancomycin were included in the treatment. First-line treatment in the UK is the second-generation fluoroquinolone, ofloxacin. The alternative first-line treatment in the UK is dual therapy, usually involving cefuroxime and gentamicin [2].The use of gentamicin drops, particularly fortified gentamicin ones (concentration 1.5%), requires caution because of the possible toxicity to the eye surface [8]. In our center, drops prepared at the pharmacy are fortified gentamicin at a concentration of 1.5%, fluconazole at a concentration of 0.02%, vancomycin at a concentration of 0.5%, and amphotericin B at a concentration of 0.5%. In the present study, all the patients diagnosed with fungal keratitis and *Acanthamoeba* keratitis required the drops prepared at the pharmacy. In total, 20% of the patients diagnosed with bacterial keratitis required these drops. Bactericidal drugs with good penetration into the eye were preferred. Fluoroquinolones have been orally and intravenously administered to patients with severe forms of bacterial keratitis. Better tolerance to fluoroquinolone drops than to reinforced drops has been reported. Moreover, both treatments have been found to have the same efficacy, with numerous side effects [9]. Gaps in fluoroquinolone coverage for *Streptococcus* and coagulase-negative *Staphylococcus* species raise concern regarding the use of monotherapy for treating bacterial keratitis [10]. In the present study, the susceptibility to fluoroquinolone was not tested in Gram-positive organisms as they are known to be highly resistant. The most important factor to be considered is the clinical response to treatment. A lack of response to treatment is the most important reason for changing the course of treatment. Dosage errors or voluntary discontinuation cannot be excluded in outpatients. The use of dual drug therapy involving ofloxacin and vancomycin as the first-line treatment for bacterial keratitis seems to be the proper solution in most cases. VRSA was not observed in our center. The use of antibiotics to which the causative pathogen is not susceptible increases the complications and cost [11].

In the present study, a decreasing trend was noted in the number of Gram-negative bacteria. The most common Gram-negative bacteria were *Serratia* spp. (24.81%), *E*.*coli* (22.55%), and *P*. *aeruginosa* (21.05%), and their numbers were similar. A decrease in the number of *Serratia* spp. and an increase in the number of *Pseudomona*s spp. were observed. The trend noted in the present study was in contrast to the downward trend in the number of *Pseudomonas* spp. noted in a previous study conducted at Manchester Royal Eye Hospital [2]. An increase in the number of cultured microorganisms may be associated with more frequent treatment of patients with keratitis who wear contact lenses. A similar trend was observed in Taiwan and China, where large numbers of patients wear contact lenses [12, 13]. In the present study, it was not possible to separate patients with keratitis into wearers and nonwearers of contact lenses because of the retrospective nature of the study. *Moraxella* was isolated as a niche Gram-negative bacterium in the local center; it was one of the main isolates in other centers [2]. However, *Moraxella* is a rare cause of keratitis (3.0%–3.9%) [13]. In the present study, 5.26% of the patients had *Moraxella* keratitis. Similar to *Pseudomonas*, *Moraxella* is more likely to be present in patients who wear contact lenses or use chronic steroid drops and in patients with diabetes [14–17]. *Moraxella* keratitis has also been found to be associated with chronic alcoholism, malnutrition, and poor sanitary habits [16, 17]. In patients with keratitis who meet the above-mentioned criteria, *Moraxella* should always be considered as the causative agent.

The most common fungal isolates belonged to *Candida* spp. and *Fusarium* spp.; they accounted for 98.25% of the total fungal isolates. *Candida* spp. and *Fusarium* spp. have also been reported to be the most common fungal isolates in previous studies [18, 19]. We observed a likely increasing trend of fungal infection, consistent with the findings of a previous study conducted in the USA [20]. However, that study focused on patients wearing contact lenses, while the present study covered the entire population with keratitis. The first-line drug has been voriconazole [2] or natamycin [21] in some studies. Our center does not routinely test for natamycin sensitivity; hence, we are unable to comment on the use of this antifungal agent. However, voriconazole appeared to have a good antifungal activity against both *Fusarium* spp. and *Candida* spp. At present, natamycin is practically unavailable in Poland. After the study in our center, voriconazole became available as the first-line antifungal drug.

*Acanthamoeba* keratitis is often culture negative and is diagnosed on the basis of clinical appearance and confocal microscopy. All the patients diagnosed in our center with *Acanthamoeba* keratitis used contact lenses. In other centers as well, a strong association has been noted with the use of lenses [2, 22–24] and with geographical variation [25]. Some cases of mixed *Acanthamoeba* and fungal keratitis have been observed [26]. The low incidence of culture-positive *Acanthamoeba* keratitis may be due to the difficulty in culturing *Acanthamoeba*.

To the best of our knowledge, this is the largest and longest analysis of the frequency, drug susceptibility, and drug resistance of pathogens causing microbial keratitis in central and Eastern Europe. In total, 27.36% of the samples were positive in the present study, while 32.6% of the samples were positive in a previous study conducted at Manchester Royal Eye Hospital [2]. A slight decreasing trend in the percentage of Gram-positive bacteria was noted. This is probably due to the fact that some patients received antibacterial and antifungal drugs before collecting the samples for microbiological tests. The main sample collected for microbiological tests was more often a conjunctival swab and less often corneal scrapes. Some of the samples were collected from patients who were finally diagnosed with noninfectious corneal ulcers, for example, peripheral ulcerative keratitis. Moreover, in some of the patients, the causative agents were nonpathogenic commensals from the ocular surface, such as *S*. *epidermidis*. This may indicate that a large number of keratitis suspected of being an infectious cause had a noninfectious cause. It should be noted that the in vitro susceptibility of bacteria may not be identical to the in vivo response [27]. On the other hand, the frequency of use, host factors, and penetration ability (in the case of eye drops) may influence the efficacy of an antibiotic [28]. Due to the retrospective nature of the present study, not all antibiotics were tested. Moreover, we were unable to investigate the change in trend in the susceptibility to some antibiotics.

An important trigger for ophthalmologists should be that the number of patients who have corneal blindness or who require evisceration/enucleation due to ocular keratitis is increasing rather than decreasing (from 0.2% to 1.0%). This is another proof that it is becoming increasingly difficult to control infectious diseases.

## 5. Conclusion

The pathogens causing keratitis change over time in terms of incidence as well as susceptibility to medications; therefore, careful monitoring and appropriate treatment adjustment are needed.

Microbiological tests assessing the susceptibility of the cultured microorganism are necessary for introducing targeted treatment. An increase in drug resistance has been observed. Implementation of the antibiotic protection program is necessary for preventing drug resistance. This situation is favored by the nonabuse of antibiotics, nonuse of antibiotics in viral inflammations, and knowledge of the current epidemiological situation in a given area, hospital, or ward, regarding the percentage of resistance of particular bacterial species to the most commonly used antibiotics. Antibiotic resistance among ocular pathogens is increasing worldwide. Resistance increases the risk of treatment failure with potentially serious consequences.

In the present study, most bacteria were sensitive to fluoroquinolones. *Ciprofloxacin* therapy remains the recommended empirical treatment in microbial keratitis. According to our study, voriconazole remains a first-line antifungal drug, when a fungal infection is suspected.

## Data Availability

The data that support the findings of this study are available from the corresponding authork upon reasonable request.

## Abbreviations

MRSA: Methicillin-resistant *Staphylococcus aureus*
VRSA: Vancomycin resistant *Staphylococcus aureus*
HLAR+: High-level aminoglycoside resistance *Enterococcus faecalis*
EUCAST: European Committee on Antimicrobial Susceptibility Testing.
